# Bone tissue regeneration: biology, strategies and interface studies

**DOI:** 10.1007/s40204-019-00125-z

**Published:** 2019-11-25

**Authors:** Mojtaba Ansari

**Affiliations:** Department of Biomedical Engineering, Meybod University, Meybod, Iran

**Keywords:** Bone regeneration, Biology, Biomaterials, Biocompatibility, Tissue engineering

## Abstract

Nowadays, bone diseases and defects as a result of trauma, cancers, infections and degenerative and inflammatory conditions are increasing. Consequently, bone repair and replacement have been developed with improvement of orthopedic technologies and biomaterials of superior properties. This review paper is intended to sum up and discuss the most relevant studies performed in the field of bone biology and bone regeneration approaches. Therefore, the bone tissue regeneration was investigated by synthetic substitutes, scaffolds incorporating active molecules, nanomedicine, cell-based products, biomimetic fibrous and nonfibrous substitutes, biomaterial-based three-dimensional (3D) cell-printing substitutes, bioactive porous polymer/inorganic composites, magnetic field and nano-scaffolds with stem cells and bone–biomaterials interface studies.

## Introduction

Bone presents numerous performs including principal backing structure designed formation, the binding place for muscles, ligaments and tendons, mechanical support and shield of most important tissues (Pajarinen et al. 2019). Besides, hematopoiesis and vital mineral materials are provided by bone marrow structure (Ansari et al. 2011). Musculoskeletal diseases including rheumatoid arthritis, osteoarthritis, osteoporosis, low back pain and limb trauma are all commonly developing and painful (Roshanbinfar and Ansari 2013). Most bone fractures occur as a result of inconvenient or incompetent bone regeneration (Naghib et al. 2012). Large segmental bone fractures did not repair instinctively and require orthopedic operation. Furthermore, spinal fusion surgeries may result in non-union frequently (Eslami et al. 2018). Approximately bone defects are currently cured using bone autograft (the gold standard for surgeons), allografts and biomaterial substitutes with biocompatible, osteointegrative “intense communications among the host bone tissue and the substituted materials”, osteoconductive “the ability of materials to be colonized by host bone cells and blood vessels” and osteoinductive “motivate host mesenchymal stem cells from encompassing tissues to differentiate to bone cells” properties (Sheikh et al. 2019). Autografts, coupled with difficulties, come across post-surgery containing nerve injury, infections, morbidity and chronic pain at the donor or acceptor site. Besides, allografts have the potential of disease conduction, infection and incite immune reactions following the implant rejection. Investigators have attempted to solve these complications of bone grafts by means of natural or synthetic biomaterials (Ansari and Eshghanmalek 2019).

This study concentrates on bone biology, bone tissue regeneration strategies including synthetic substitutes alone, scaffolds combined with active molecules, nanomedicine for healing of bone trauma and defects, cell-based combination products with cells from various sources, biomimetic fibrous and nonfibrous substitutes, biomaterial-based three-dimensional (3D) cell-printing substitutes, bioactive porous polymer/inorganic composite and magnetic field and nano-scaffolds with stem cells, bone biomaterial interface studies. The interactions between bone cells and biomaterials, as they play a crucial role in bone repair.

## Bone biology

### Bone structure

Bone is not homogeneously solid, but it is arranged of living bone cells set in a biomineral medium. Actually, bone is designed by the toughening of this medium nearby entangled cells. Bone itself involves chiefly of collagen fibers and an inorganic bone mineral in the form of small crystals (Uskokovic et al. 2019).

### Bone cells and matrix

The biomineral medium of bone contains about 30% organic and 70% inorganic segments (Wang et al. 2019). Almost, 90% of this organic segment is collagen, whereas the residual 10% was mostly non-collagenous proteins, lipids, proteoglycan molecules, osteopontin (OPN), and other bone matrix proteins (Hu et al. 2019). The bone matrix proteins play vital role in mechanical strength and tissue adhesive characteristics. Principally, the mineral phase of bone is hexagonal hydroxyapatite (HA) crystal (Qiu et al. 2019). The chemical formula of crystalline HA is Ca_10_(PO_4_)_6_(OH)_2_ (Türk et al. 2019), where surface binding and electrostatic interactions are related to presence of Ca^2+^ and (PO_4_)^3−^ (Samavedi et al. 2013). The HA crystals are organized parallel to the long axes of collagen fibers by self-assembly of collagen triple helices (Wang et al. 2012).

#### Bone cells

**Fig. 1:**
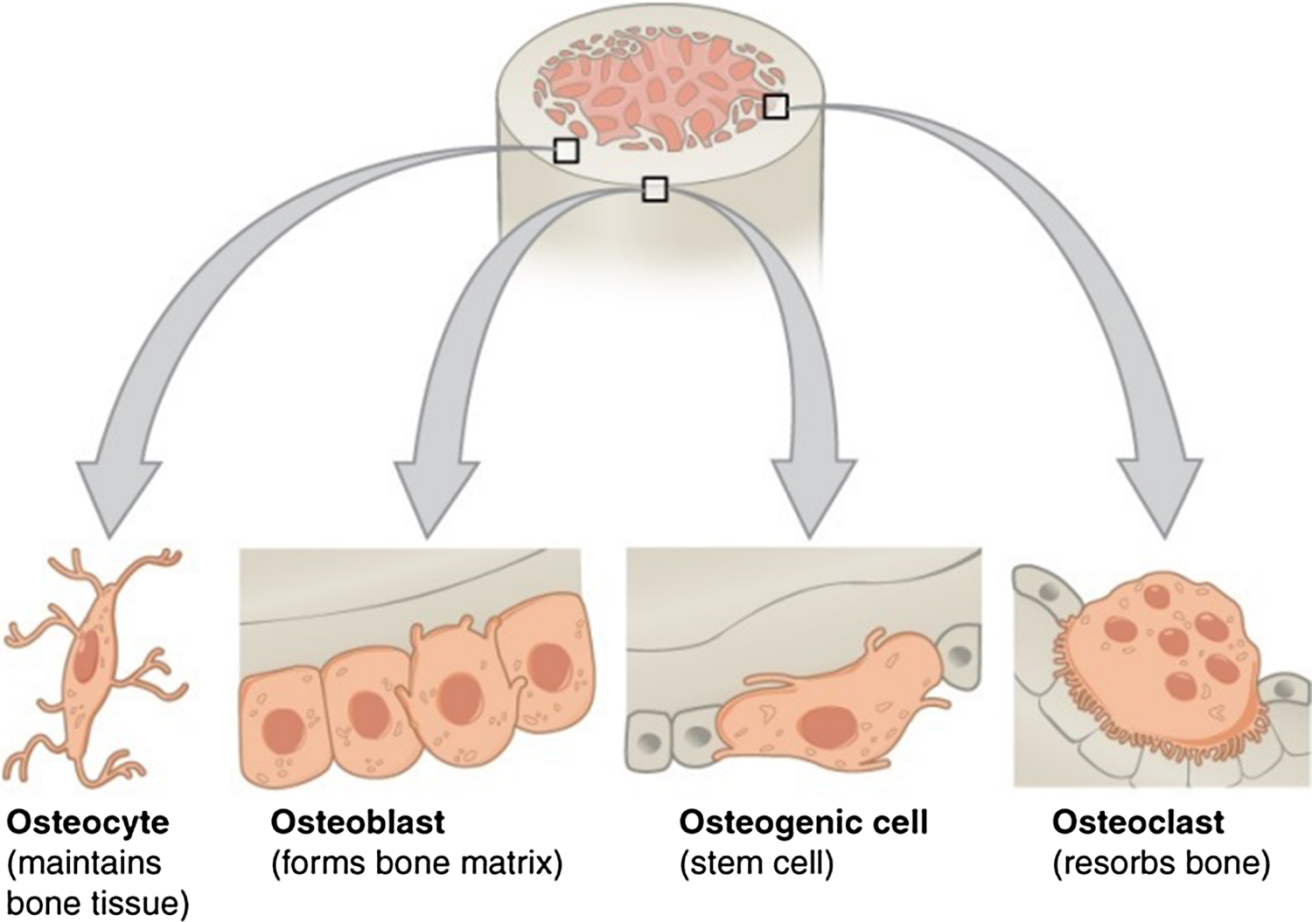
Four types of cells are found within bone tissue including osteogenic cells, osteocytes, osteoclasts, and osteoblast (Behzadi et al. 2017)

Further to mineralized bone milieus, bone cells are additionally critical to the function of bones. Bone is responsible for several roles in the body containing mechanical functions (protection, shape, movement and locomotion), synthetic functions (synthesis of blood cells) and metabolic functions (mineral storage, regulation of calcium and phosphate, fat storage and role in acid–base balance). The four most important cells including: osteogenic, osteoblasts, osteocytes, and osteoclasts (together recognized as the basic multicellular unit (BMU)) contained in bone regeneration and structure are shown in Fig. 1 (Kular et al. 2012).

Osteoblasts are derived from osteoprogenitor cells of mesenchymal origin in bone marrow and other connective tissues. They are differentiated and proliferated to osteoblasts before bone formation, stimulated through bone morphogenetic proteins (BMPs). These cells are responsible for growing or remodeling of bone (Standring 2016). Also, their roles are the synthesis, deposition and mineralization of the bone matrix by producing a protein mixture called osteoid. Mature osteoblasts may convert to a layer of cuboidal cells, that they can undergo apoptosis or become osteocytes and bone-lining cells (Sikavitsas et al. 2001).

Osteocytes are the most abundant cell type in bone tissue (Noble and Reeve 2000). They are described by a star-shaped morphology. Osteocytes are derived from MSCs that undergo osteoblastic differentiation. These are inactive osteoblasts that have become trapped in the bone that they have created. They maintain connections to other osteocytes and osteoblasts. They are vital for communication within bone tissue. Furthermore, osteocytes have been presented to react to several biochemical signaling paths and contribute to regulation of calcium and phosphate homeostasis. Malfunction of the osteocyte cells growths leads to bone brittleness and may result in osteoporosis (Standring 2016; Teti 2011).

Bone-lining cells are inactive osteoblasts that reside on bony surfaces (Franz Odendaal et al. 2006). Lining cells play a significant role in coupling bone resorption to bone formation and in calcium hemostasis and in osteoclastic differentiation. They also act as hurdle that avoids direct interface among osteoclasts and bone matrix (Luginbuehl et al. 2004).

Osteoclasts are large cells with more than one nucleus that are differentiated from the hematopoietic lineage. Their job is to break down bone (Boyle et al. 2003). They release enzymes and acids to dissolve minerals in bone and digest them. This process is called resorption. Osteoclasts help remodel injured bones and create pathways for nerves and blood vessels to travel through. Irregularities in osteoclastic activity distinguish diseases such as osteoporosis (increased osteoclast activity) and osteopetrosis (Kular et al. 2012; Standring 2016).

### Mechanism of bone repair

**Fig. 2:**
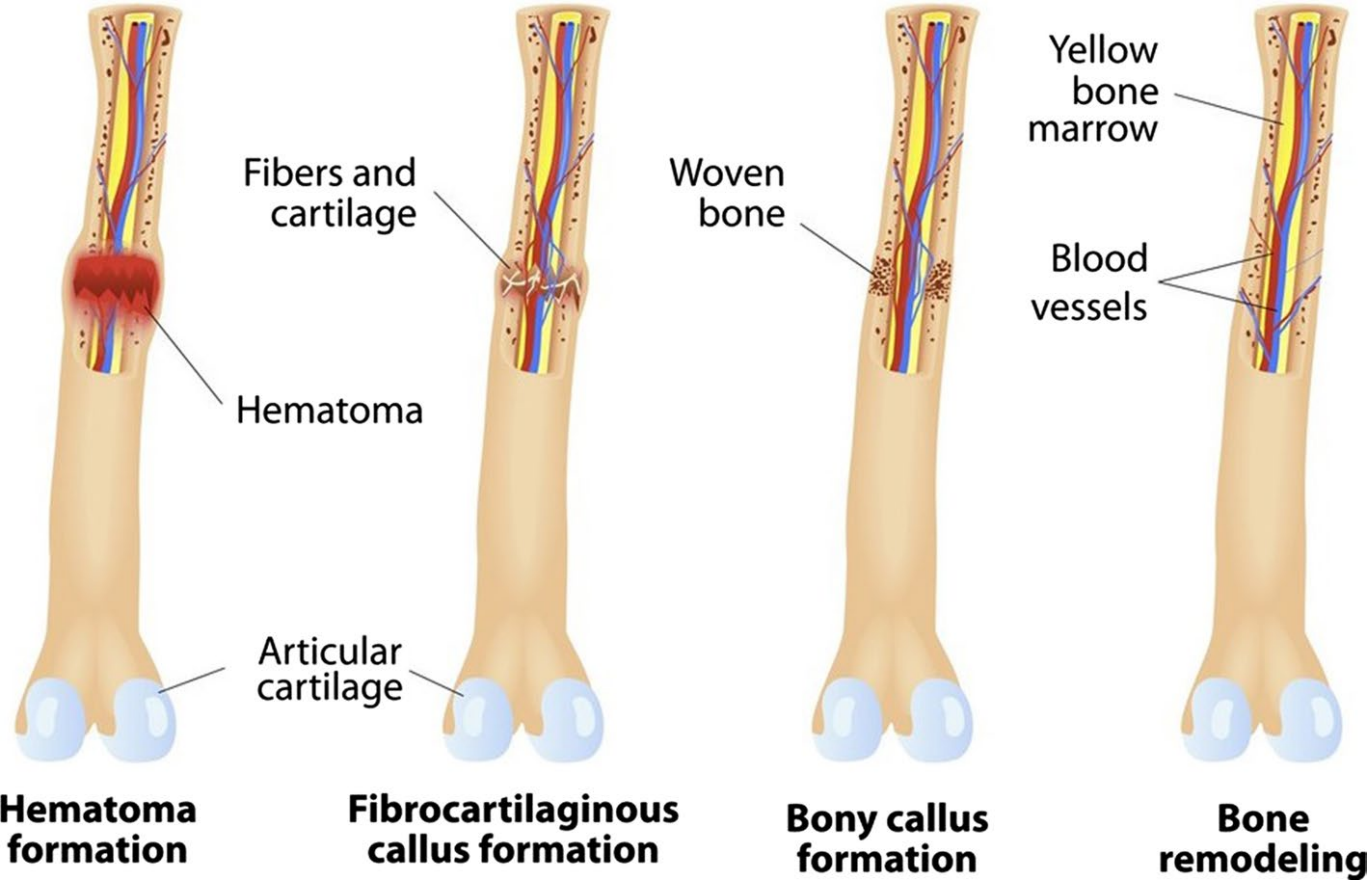
Stages of bone fracture repair and remodeling

Bone fracture regeneration is a multipart, arranged, reformative procedure that contains a vital numeral of progenitor cells along with inflammatory, endothelial and hematopoietic cells. The bone restorative procedure has three intersecting phases: inflammation, bone production and bone remodeling (Schindeler et al. 2008). Inflammation begins instantly once the bone is broken and continues for more than a few days. As soon as the bone is broken, there is bleeding into the region, result in inflammation and coagulation of blood at the breakage location (Sikavitsas et al. 2001). This is responsible for the primary fundamental strength and basis for new bone formation. Bone production initiates once the coagulated blood formed by inflammation is substituted with fibrous tissue and cartilage (recognized as soft callus). As regeneration growths, the soft callus is switched with hard bone (identified as hard callus), which is noticeable on X-rays some weeks after the fracture. Bone remodeling, the ultimate stage of bone healing, continue more than a few months. In regeneration, bone regenerates to form and converts condensed, returning to its original form (Dimitriou et al. 2005). Furthermore, blood circulation in the region progresses. Once suitable bone restorative has followed, weight bearing inspires bone healing. Briefly, the stages of bone fracture repair are presented in Fig. 2.

## Bone regeneration strategies

At present, the “conventional standard” healing of patients suffering from long or imperfect bone treatment is to implement bone grafting, by means of either an autograft or an allograft. Though, there are problems to bone grafting. Subsequently, a more maintainable, long-term healing plan is necessary. To that end, bone graft replacements are being concocted to aid damaged fracture treatment. Based on the gravity of the trauma, the main strategies are established for bone repair:

1. Synthetic substitutes alone
2. Scaffolds combined with active molecules
3. Nanomedicine for healing of bone trauma and defects
4. Cell-based combination products with cells from various sources
5. Biomimetic fibrous and nonfibrous substitutes
6. Biomaterial-based 3D cell-printing substitutes
7. Bioactive porous polymer/inorganic composite
8. Magnetic field and nano-scaffolds with stem cells.

### Suitable material selection of damaged bone substitute

Demineralized bone matrix (DBM) was produced with osteoconductive and osteoinductive properties (Giannoudis et al. 2005). The preferred choice for DBM synthesis was reported cortical bone as a result of osteoinductive with a lesser antigenic possibility in comparison to cancellous bone (Burg et al. 2000). Utilization of viscous spongy cellulose revealed that it was extremely suitable for bone regeneration. The implanted cellulose in the femoral bone of rats showed that osteoconduction was mostly happened (Ekholm et al. 2005). Recently, bacteria strains secretion-derived bacterial cellulose was introduced as an emerging player in tissue engineering because of its extremely good cytocompatibility and physiochemical properties. Therefore, its modified compounds were used for bone regeneration (Stumpf et al. 2018). Synthetic media have similarly been evaluated as acellular bone tissue engineering materials. Hence, poly-l-lactide (PLLA) films were used to repair 1-cm trauma in the radius bone of mature rabbits, and histologic results indicated that the cortical bone was redeveloped over the defect (Zhang et al. 2006). Poly-ε-caprolactone-*co*-lactide was used as a different potential filler material in bone defect, and investigated in non-osseous usage. To evaluate the absorption and biocompatibility of this copolymer, it was used in femoral defect of rat (Helminen et al. 2002). Photocrosslinkable polyanhydrides constituents demonstrated the convinced benefits for orthopedic regeneration. In this case, the photopolymerizable component enhanced microfabrication probability of porous scaffolds. Also, mechanical investigations proved reliability of these polymers for tissue engineering applications (Pakulska 2016). Calcium phosphate and silicate-based bioceramics were prominently featured among used biomaterials for bone regeneration (Samavedi et al. 2013; Diba et al. 2014; Dziadek et al. 2017). For example, nanostructured monticellite (CaMgSiO_4_) ceramic and its composites (with HA) showed good in vitro bioactivity, biocompatibility, and antibacterial properties for bone tissue engineering application (Chen et al. 2008; Kalantari et al. 2017; Kalantari et al. 2018a, b, c, 2019; Kalantari and Naghib 2019).

### Scaffolds combined with active biomolecules

**Fig. 3:**
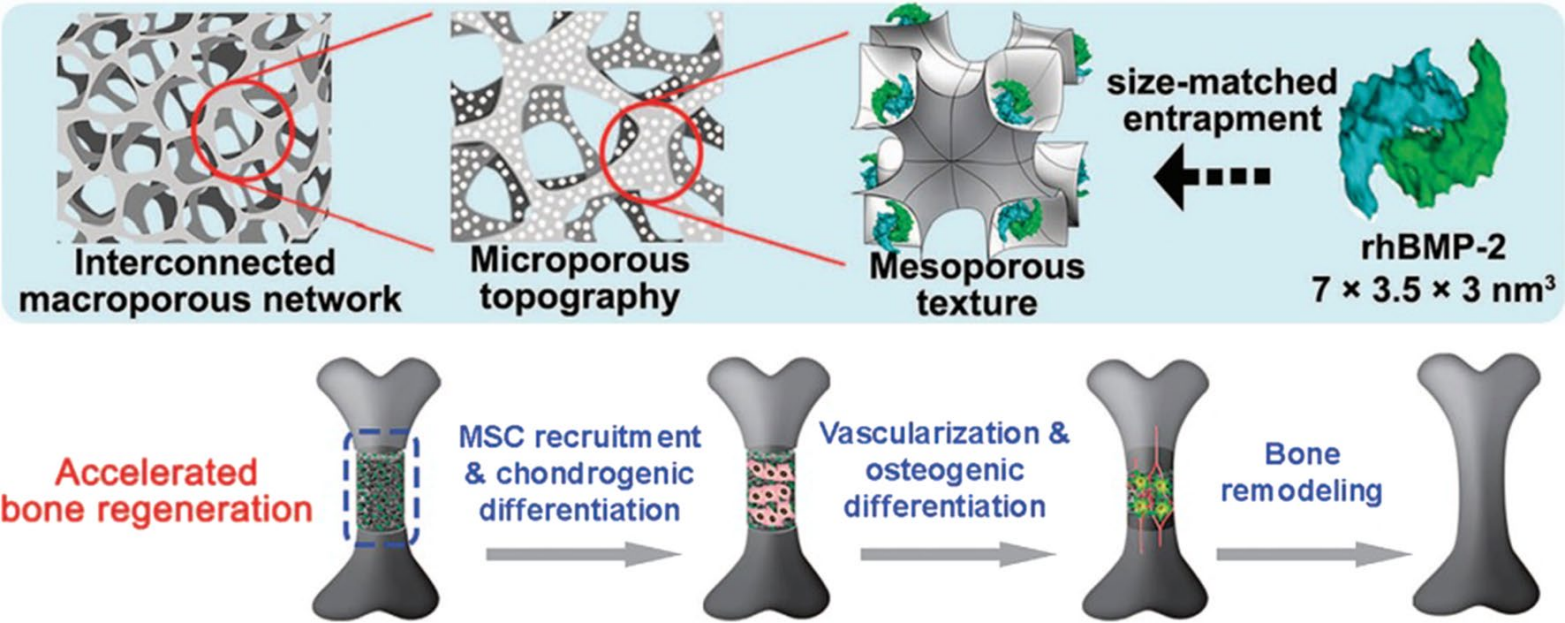
Schematic presentation of macro/micro/nano-porous scaffold loaded with active biomolecules for accelerate bone regeneration (Yi et al. 2016)

The approaches can be categorized in three classifications: (a) application of recombinant growth factors and a combination of growth factors associated with a natural medium or calcium phosphate substantial carrier, (b) application of proteins to target cellular receptors, and (c) application of small molecules that target the signaling pathway (Ansari et al. 2018). Figure 3 presents the general schematic presentation of the scaffold loaded with active biomolecules for accelerate bone repair. The main growth factors have already been used in clinics included BMP-2, BMP-7, and rhPGDF-BB (Ho-Shui-Ling et al. 2018). The growth factors effect on bone progenitors by interrelating with their particular receptors, which activate the chemical signaling pathways result in bone development. Several studies have previously been done on BMP-2 associated with a type I collagen porous structure as delivery service, for application in open tibial fractures and in spinal defects (Bessa et al. 2008). In a study, it is combined with a titanium or PEEK structure for application in anterior lumbar interbody fusion (Vaidya et al. 2008). It is well known that bone restoration in response to BMP-2 is dose dependent. High doses of BMP-2 may result in osteolysis. So, the potent activating characteristic of bone repair of BMP-2 needs to be further optimized (Bruder et al. 1994). Latest documents in a rat femoral bone fracture, applied PLGA as a polymeric carrier as a nano reservoir for BMP-2 delivery (Zheng et al. 2010). The result revealed that it is conceivable to adjust the dose of BMP-2 in vivo. This controlled dose influenced the dimensions of bone formation, and enhanced the kinetics of bone renovation by means of delivery of BMP-2.

BMP-7 growth factor is osteoinductive which is studied for the clinical application (Bostrom and Seigerman 2005). Collagen type I incorporated with BMP-7 in the paste form is used for recalcitrant long bone and spine surgical treatment. Investigations on a sheep model indicated that the BMP-7 paste may be able to incorporate with a porous scaffold to initiate long bones regeneration (Haidar et al. 2009). rhPDGF-BB in form of device/drug product was employed for hindfoot and ankle fusion (Solchaga et al. 2012). PDGF, by functioning on PDGF receptors, motivates the employment, migration and proliferation of cells containing mesenchymal stem cells and stimulates the neovascularization by growing vascular endothelial cells at the location of bone repair.

Presently, a β-TCP/PDGF scaffold was employed to provide osteoconductivity for bone repair. In a study on patients devoted to hindfoot or ankle arthrodesis, healed with rhPDGF-BB/β-TCP, gave rise to comparable fusion extents, minus pain, and less side effects in comparison to healing with autograft (Bateman et al. 2005).

Other growth factors like GDF-5 are kind of BMP group that encourages bone formation (Nickel et al. 2005). Several researches have confirmed rhGDF-5 has the potential of bone induction tissue growth. A bone substitute rhGDF-5/(β-TCP) was applied for dental implants and medical cure of periodontal syndrome. The in vitro results indicating that rhGDF-5 has the potential to promote gene expression and production of the ECM proteins such as collagen type II and aggrecan (Poehling et al. 2006).

Peptides, they have the ability to access cellular receptors, are substitutes for recombinant growth factors which are able to produce easily. Bioactive B2A (B2A2-K-NS) synthetic polypeptide applied to augment spinal fusion (Omrani et al. 2016). HAP/β-TCP incorporated with B2A granules were investigated for foot and ankle fusion.

In vitro results indicate that B2A induces chondrogenic differentiation and improves the in vivo healing of injured cartilage in an osteoarthritis model (Ho-Shui-Ling et al. 2018). Collagen is a major protein of the ECM and contributes in osteoblast attachment and activity (Ansari and Moztarzadeh 2012). P-15 is a 15 amino-acid protein obtained from collagen and promotes the differentiation of mesenchymal stem cells. P-15 has been applied in combination with bone inorganic for spinal fusion, non-union fractures and joint reconstruction with acceptable results (Bhatnagar et al. 1999). Besides, small molecules applied as controllers of bone bulk. Parathyroid hormone has a vital character in controlling calcium phosphate digestion. PTH entrapped in a normal fibrin medium, incorporated with a mechanical ceramic constituent (HAP/TCP granules), may be responsible for structural stability and osteoconduction for the period of healing (Portale et al. 1984).

Microorganism-derived polyhydroxyalkanoate (PHA) scaffolds have emerged as polymeric promising biomaterials with excellent potential for bone tissue engineering applications because of their good biodegradability, biocompatibility and vascularization, and unique physiochemical properties. They induced cell adhesion and growth on their porous structure for bone regeneration (Lim et al. 2017). Lalzawmliana et al. (2019) reported that mesoporous bioactive glass (MGB) scaffolds as third-generation biomaterials were used for regeneration of critical bone defects, and MGB scaffolds should have large interconnected pores for improving growth, adhesion and proliferation of osteoblast cells and assisting in angiogenesis. In recent studies, effect of three-dimensional (3D) scaffolds and their fibrous order on biocompatibility were investigated. The results showed that 3D aligned nanofibrous scaffolds provided cell behaviors better than two-dimensional (2D) scaffolds, because of their more accommodation for the attached cells, and loading more bioactive molecules for promotion of cell growth, proliferation, migration and differentiation. Nevertheless, a big challenge was mentioned for them that related to their static status. For example, they cannot remodel their stiffness, surface chemistry and roughness in an in situ and dynamic situation, so cannot mimic the function of main tissue (Jin et al. 2018). Rather et al. reviewed the dual functional strategies to spread osteogenesis coupled angiogenesis through different scaffolds. Vascularization played the important role to carry oxygen, nutrients and essential molecules and growth factors into damaged tissue. Then, the angiogenesis and osteogenesis communicated harmoniously together for bone regeneration, because many studies confirmed the crosstalk between bone progenitor cells and endothelial cells. Therefore, the scaffolds containing osteoinductive and angioinductive factors released various types of molecules to stimulate osteogenesis and angiogenesis (Rather et al. 2019). The studies showed that many scaffolds were investigated in the field of bone repair with the purpose of bettering cell growth, adhesion and proliferation, osteogenic differentiation, vascularization, and mechanical properties, but Roseti et al. (2017) reported that the further depth studies will be needed for using the bone tissue engineering scaffolds in clinical application.

### Nanomedicine for healing of bone trauma and defects

One of the most important drawbacks in the healing of open fractures is infection. The failure of the tissue obstruction among the rupture location and the external milieu result in bone bacteriological infection and contamination (Broos and Sermon 2004).

Besides, it was proved that *Staphylococcus aureus* (is a Gram-positive, round-shaped bacterium that is a member of the firmicutes, and it is a usual member of the microbiota of the body) may attack intracellular sites contained by osteoblasts, result in complications in microbial eradication and amplified vulnerability to osteomyelitis subsequent contamination (Join Lambert et al. 2005). The infected fractures need management including medical debridement, antibiotics, and skeletal stabilization. Frequently, antibiotic-contained cement made of polymethylmethacrylate (PMMA) is implanted to harmonize the antibacterial activity (Schade and Roukis 2010). The growth and differentiation of osteoblasts and osteoclasts are controlled by growth factors, cytokines produced in the bone-marrow ECM, and adhesion structures that facilitate cell–cell and cell–ECM communications (Lu et al. 2012).

**Fig. 4:**
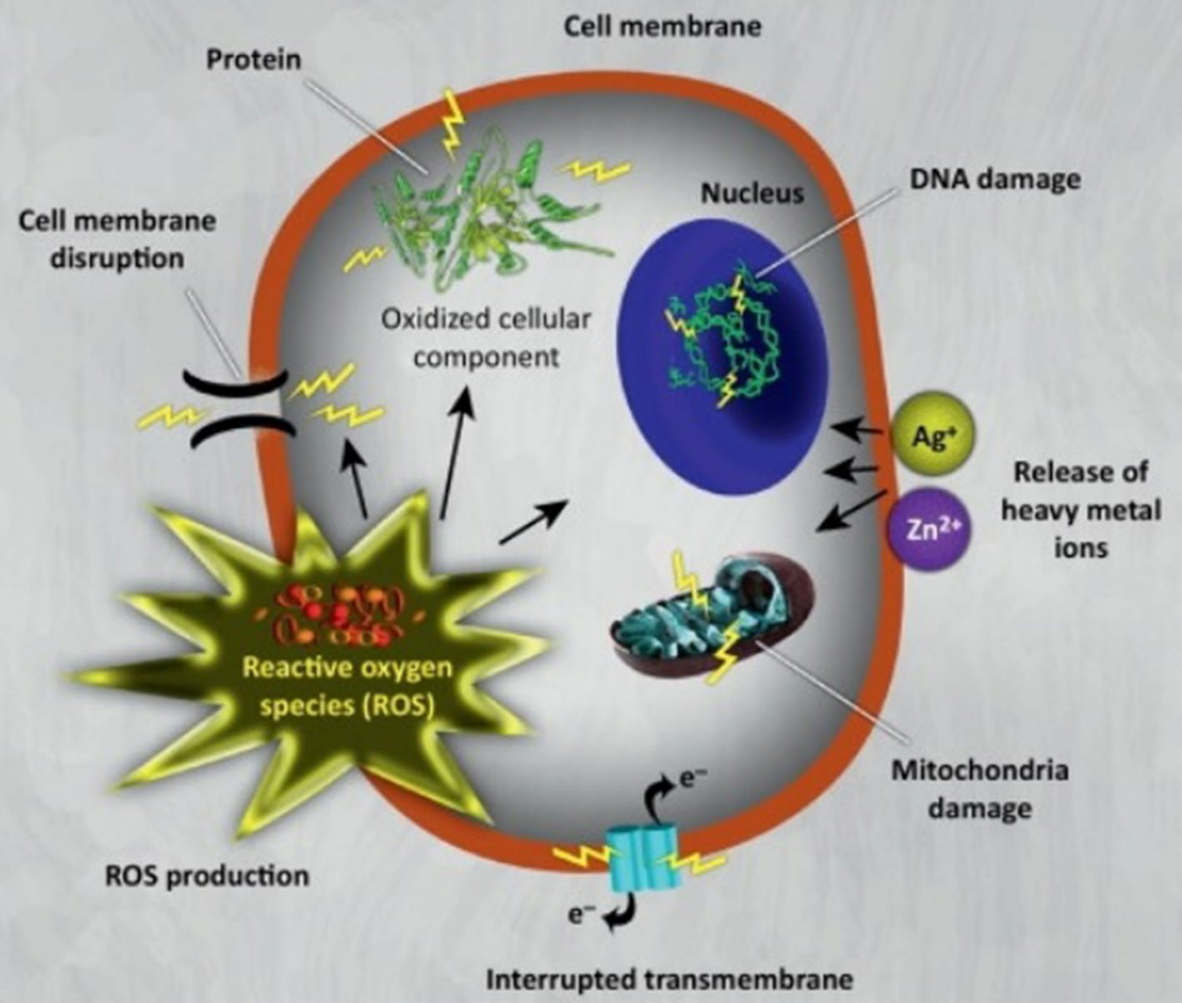
Toxicity mechanisms of NPs and their ions (e.g., silver and zinc) against bacteria by induce oxidative stress by means of the production of reactive oxygen species (ROS). The ROS is able to conclusively break bacteria (e.g., their membrane, DNA, and mitochondria) culminating in bacterial death (Hajipour et al. 2012)

Numerous categories of antibacterial nanoparticles (NPs) and nano-sized carriers for antibiotic delivery have been confirmed to be applicable in curing infectious diseases, containing antibiotic-resistant ones, in vitro and in vivo. For instance, several NPs are able to join to the membrane of microorganisms by electrostatic interface and destruct the unity of the microorganism membrane (Banerjee et al. 2011). Another mechanism is that designed NPs are able to produce massive oxidative stress to microorganisms through free radical construction such as reactive oxygen species (ROS) and destroy their contamination hazard as it is shown in Fig. 4 (Long et al. 2006).

One of the most frequently used NPs for diminution of infection risk in orthopedic distress is silver (Ag) NPs. Their extensive antibacterial performance is proved. They are extensively applied to remove various bacteria containing *S. aureus*, *Bacillus subtilis*, *Klebsiella pneumoniae*, *Pseudomonas aeruginosa*, and *Escherichia coli*.

Polymethylmethacrylate (PMMA)-based bone cement, consisting of Ag NPs (about 50 nm), completely prevented the propagation of *Staphylococcus epidermidis*, methicillin-resistant *S*. *epidermidis* (MRSE), and methicillin-resistant *S. aureus* (MRSA), while PMMA bone cement contained 2% of gentamicin sulfate avoided only the propagation of *S. epidermidis* (Abid et al. 2017). In additional research, an antibacterial HA NPs scaffold was created, and antibacterial properties were attained by the addition of Ag NPs.

In another study, selenium (Se) NPs were employed to coat a bioactive glass-based structure (SiO_2_ and molar ratio of P to Ca = 1/5) fabricated by the foam replica technique. The results indicated that this scaffold has antibacterial activity (Fathi-Achachelouei et al. 2019)..

### Cell-based combination products with cells from various sources

Tissue-particular cells such as osteoblasts maybe employed as the cellular constituent of bone transplants. Several kinds of stem cells have been mostly used through the construction of bone grafts (Omrani et al. 2019). Multipotent adult mesenchymal stem cells (MSCs) show unlimited differentiation capacity into various kinds of cell lineages, containing osteoblasts and chondrocytes (Baksh et al. 2004). Adult MSCs perform as an inducible backup potency for tissue restoration following damage, and have been considered broadly for bone fracture regeneration. MSCs obtained from numerous diverse sources containing bone marrow, synovial membrane, skeletal muscle, and adipose tissue. Cell-based therapy with allogenic BMSCs implants is operative in bone regeneration in different animal bone defect models. In initial clinical studies, autologous BMSCs have been cultured on bio-ceramic scaffold to heal big bone defects. Local transplantation at the defect situate of MSCs led to widespread fusion at 5–7 months after surgery (Cancedda et al. 2003).

Another study indicated bone repair in rabbit skull defects healed with autologous, osteogenically induced adipose-derived stem cells (ADSCs) transplanted onto fibronectin-coated polylactic acid scaffold (Di Bella et al. 2008). Additional study showed cranial bone defect regeneration in canine by means of osteogenically induced ADSCs transplanted onto a coral structure (Aimaiti et al. 2011). In a different research, calvarial defects treated through autologous ADSCs/fibrin glue/autologous cancellous bone graft. After 2 months, new bone mineralization and complete calvarial integrity were observed (Lendeckel et al. 2004).

Multipotential synovial membrane-derived MSCs (SMSCs) stromal cells can operate as a healing substitute for focal cartilage damages and have capability to differentiate to osteogenesis. Stimulatingly in a current research, SMSCs from knee joints presented greater osteogenic and adipogenic potential than SMSCs of hip joints (Kristjánsson et al. 2013).

Dental pulp-derived stem cells (DPSCs) have lately being discovered for bone tissue engineering. DPSCs present the low percentages of morbidity, widespread differentiation capacity into chondrogenic, and osteogenic cell lines, and expression of bone markers in vitro and in vivo (d’Aquino et al. 2008). Alginate microsphere DPSCs carrier has the osteogenic potential through detecting improved mineralization and upregulated intensities of osteogenic genes (Moshaverinia et al. 2012). DPSCs seeded/collagen-HA-poly(l-lactide-*co*-ɛ-caprolactone) scaffold confirmed effective ECM mineralization of osteoblast (Akkouch et al. 2014). Some tissues for instance placenta, umbilical cord blood (UCB) and umbilical cord tissue are different sources of MSCs (Jin et al. 2013). The regenerative capability of RGD-functionalized microporous calcium phosphate cements (CPC) contained UC MSCs and BM MSCs were compared in a rat bone defect model. The results showed comparable great bone inorganic compactness, new bone formation and vascularization in vitro and in vivo (Gan et al. 2018).

Pluripotent human embryonic stem cells (hESCs) are obtained from human blastocysts. Effective differentiation of hESCs into the osteogenic lineage has been confirmed in frequent reports mutually in vitro and in vivo. Actually, following osteogenic stimulation, hESCs demonstrated to retain molecular and fundamental characteristics similar to bone cells by means of the creation of mineralized bone in vitro. Osteogenic cells derived from ESCs seeded on poly(d,l-lactic-*co*-glycolic acid)/HA scaffold showed substantial in vivo bone construction in immunodeficient mice through subcutaneously seeding (Tang et al. 2012).

Induced pluripotent stem cells (iPSCs) have been originated from adult somatic cells such as skin fibroblast (Kim et al. 2010). IPSCs have the capacity to differentiate to all cell types. IPSCs obtained from embryonic source have the ability to produce MSC-like cells in vitro which presented the capability of more differentiating property to osteoblast cells, whereas similarly indicating osteogenic capacity comparable to that of BMSCs in vivo. Furthermore, in vivo investigations have confirmed that MSC-like cells obtained from iPSCs present the capability to develop mature mineralized construction similar to bone structure (Wu et al. 2017).

Endochondral bone tissue engineering using progenitor cells such as chondroprogenitors has been lately demonstrated. Several researches presented that articular chondrocytes are able to be stimulated to endochondral ossification and generate TGFβ-1 and BMP-2 (Perez et al. 2018). In a study, chondrocyte cell seeded on BMP-2-loaded polycaprolactone (PCL) scaffold which subcutaneously implanted in vivo result in bone formation (Lee and Shin 2007).

### Biomimetic fibrous and nonfibrous substitutes

Bone tissue has a mineralized construction. Biomimetic composite substitute with a mineral constituent were used broadly for bone repair. The mineral component induces structural integrity and osteoconductive properties to the scaffold. HA is frequently used for the reason that has the potential to simulate the natural minerals part of bone. Besides, other calcium phosphate or bioglass were similarly used for their biocompatibility. Using dioxane/water as a solvent, nano-HA/PLLA nanofibers composite scaffolds through TIPS (thermally induced phase separation) technique were fabricated. The high surface area of the nanofibrous permits further the HA to be exposed, which is appropriate for bone tissue regeneration (He et al. 2009).

In another study, HA was incorporated into electrospun nanofibers, then utilized a gelatin-apatite precipitate homogenized in an organic solvent with polylactide-*co*-caprolactone (PLCL). For the duration of the precipitation reaction, the Ca/P proportion was reserved to 1.67 to guarantee stoichiometric apatite fabrication. Just the lowest concentration of gelatin-apatite leads to a growth in normal strength (Kim et al. 2006).

Lately, electrodeposition method has been developed that decreases the mineralization time. To demonstrate the flexibility of the technique, electrodeposition has been effectively made on both electrospun PLLA fibers and phase-separated PLLA fibers. Consequently, electrodeposition confirmed to be a fast and operative method to mineralize a bone tissue scaffold (Wei and Ma 2006). Collagen, in the form of injectable hydrogels, membranes, or sponges, extensively employed for bone tissue regeneration. Individually, as composite with calcium phosphate structures such as HA; Several instances include, collagen/HA/chitosan or collagen/HA/alginate hydrogels (Teng et al. 2008).

### Biomaterial-based 3D cell-printing substitutes

3D printing employs 3D images of the bone trauma anatomy, usually acquired from computed tomography (CT) scans, using a calculating software, to fabricate a bone graft substitutes (BGS) structure that matches to a bony defect (Burleson and DiPaola 2019). The personalized bone graft substitute form is fabricated using a 3D printer to control the BGS mechanical features and substantial parameters. The composition optimization confirms an improved correspondence among the BGS and the patient’s anatomy, permitting the regeneration. Metallic replacements manufactured by titanium are the further most extensively used. Titanium plates are usually employed to immobilize bone parts in jaw operations. 3D printing is similarly being studied for orthopedic purposes: for acetabular ruptures, ankle defects and further bone defects due to bone fracture, spurt fissure of spine, bone cancer and orbital ground repair. The tailored spongy implant printed using Ti_6_Al_4_V presented outstanding physicochemical features and biological function such as biocompatibility, osteogenic property, and bone regeneration (Alvarez and Nakajima 2009). Bioceramics and biopolymers such as polyetheretherketone (PEEK) are currently custom designed, and are presently being investigated at the pre-clinical phase (Yan et al. 2015). PCL/HA composite is being studied for the repair of gingival recession concomitant with bone and gingival tissue repair (Osathanon et al. 2017).

**Fig. 5:**
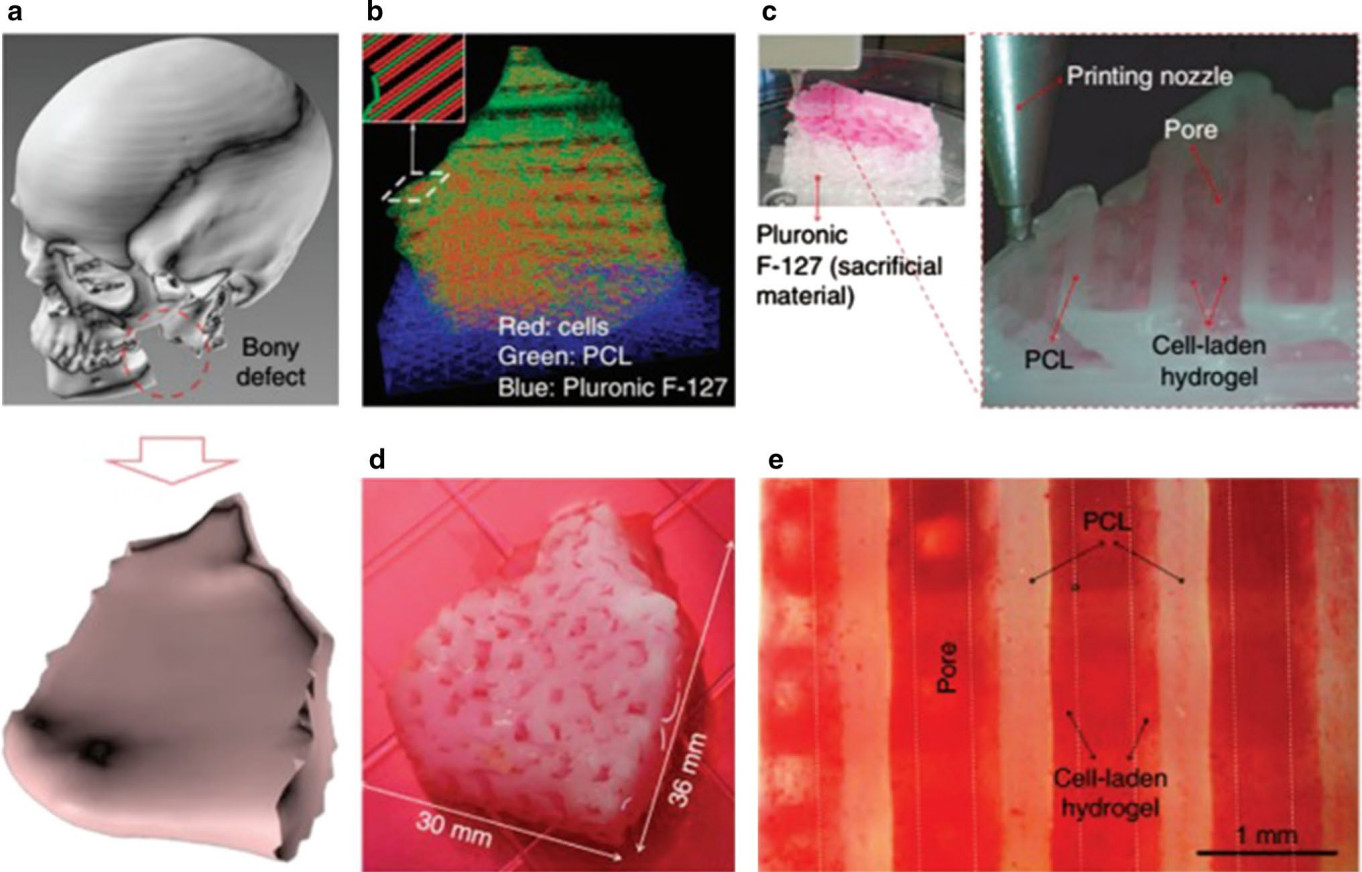
Mandible bone regeneration. **a** 3D CAD model identified a mandible bony defect from human CT image data. **b** Visualized motion program was generated to construct a 3D architecture of the mandible bone defect using CAM software. **c** 3D printing process using integrated organ printing system. **d** Photograph of the 3D-printed mandible bone defect construct, which was cultured in osteogenic medium for 28 days. **e** Osteogenic differentiation of hAFSCs in the printed construct was confirmed by Alizarin Red S staining, indicating calcium deposition (Jang et al. 2018)

In a recent study, a mandible bone was repaired via human amniotic fluid**-**derived stem cell (hAFSC)-laden hydrogel, a mixture of PCL and tricalcium phosphate **(**TCP), and pluronic F127 (Fig. 5b). The PCL/TCP and hAFSCs mixed with the combination of hydrogel were reproduced in a type I design with a Pluronic F127 impermanent support (Fig. 5c). Subsequently induction of osteogenic differentiation for 28 days (Fig. 5d), they stained the constructions with Alizarin Red S; staining at the surface of the 3D bone constructions showed calcium deposition in the hAFSC laden hydrogel (Fig. 5e).

### Bioactive porous polymer/inorganic composites

The artificial and biodegradable, polymer/inorganic bioactive part compounds are used as bone tissue engineering supports as a result of their formability, bioactive performance and regulating biodegradation kinetics (Rezwan et al. 2006).

Two categories of biodegradable biopolymers are presented: the natural polymers containing polysaccharides such as starch, alginate, chitin/chitosan, hyaluronic acid, proteins for instance collagen, fibrin gels, silk and, as reinforcement, a diversity of bio-fibers including lignocellulosic natural fibers, and the synthetic polymers are used as 3D scaffolds in bone tissue engineering, are saturated poly-α-hydroxy esters, containing polylactic acid (PLA) and poly glycolic acid (PGA), as well as polylactic-*co*-glycolide (PLGA) copolymers (Gentile et al. 2014).

PPF (polypropylene fumarate) has been used as an injectable bone substitute scaffold for conducted tissue regeneration. It was similarly utilized as a substrate for osteoblast cultures. The growth of composite substrates adjoining polypropylene fumarate and mineral elements, such as HA or bioglasses, in contrast with the broad investigation works devoted to PLGA and PLA composites (Chen et al. 2012).

Aliphatic polyesters PHAs manufactured through bacteria under unstable progress situations. They are commonly biodegradable (through hydrolysis), biocompatible and thermoprocessable (Lizarraga-Valderrama et al. 2016). These fascinating properties make them suitable for biomedical applications in particular tissue engineering. PHA, principally poly-3-hydroxybutyrate (PHB), copolymers of 3-hydroxybutyrate and 3 hydroxyvalerate (PHBV), poly-4-hydroxybutyrate (P4HB), copolymers of 3-hydroxybutyrate and 3-hydroxyhexanoate (PHBHHx) and poly-3-hydroxyoctanoate (PHO) were confirmed to be appropriate for bone tissue regeneration (Ke et al. 2017).

Degradation products of bioglasses, especially the 45S5 Bioglass structure, regulate the gene activation that manages osteogenesis and the fabrication of growth factors (Xynos et al. 2001). HA and silicon have a vital character in the bone mineralization and gene expression, which requires greater than before attention in the substitution of silicon for calcium into HA structure (Arvidson et al. 2011). In vivo results have revealed that bone remineralization into silicon-doped HA particles has been significant larger than that pure HA. Bioactive glasses lately have been used as scaffold, filler or coatings of polymers and, as porous constituents, which contains melt-derived and sol–gel-derived bioglasses (Wang and Yeung 2017).

In vivo and in vitro evaluation of crystalline or amorphous calcium phosphates, in bulk, coating, powder, or porous form, induce the attachment, differentiation, and proliferation of osteoblasts and mesenchymal stem cells (Rezwan et al. 2006).

### Magnetic field and nano-scaffolds with stem cells

Innovative approaches are using magnetic nanoparticles (MNPs) and magnetic fields to improve bone repair efficiency containing osteogenic improvements by means of magnetic fields, MNPs and magnetic approaches to develop the cells, scaffolds and growth factor conveyances including cell tagging, targeting, designing, and gene modifications (Panseri et al. 2012). The process of scaffolds containing MNPs using magnetic fields and stem cells to improve bone redevelopment were recognized as including the motivation of signaling trails containing MAPK, integrin, BMP and NF-κB (Gonçalves et al. 2016). Static magnetic fields (SMFs), pulsed electromagnetic fields (PEMFs), rotating magnetic fields (RMFs) and alternating electromagnetic fields have the potential to improve the defect healing, bone mineral density, attachment of implants among bone tissue (Xia et al. 2018b). Combination of magnetic fields with growth factors and signaling factor, magnetically aided freezing and defrosting of stem cells, magnetically aided scaffold and coating constructions are able to improve bone restoration. Animal research presented that SMFs with moderate intensity improved the bone mineral compactness and bone repair (Fitzsimmons et al. 1995).

SMFs may possibly modify cell functions such as the attachment, morphology, proliferation, differentiation, apoptosis, gene expression, in particular osteogenic differentiation for different kinds of cells, containing BMSCs, human osteosarcoma cell lines MG63, human adipose-derived MSCs, and dental pulp stem cells (DPSCs) due to electrodynamic interactions and magneto mechanical interactions (Xia et al. 2018b). Superparamagnetic iron oxide nanoparticles (SPIONs) are encouraging for targeted drug delivery, tissue engineering, hyperthermia, gene therapy, imaging and cell tracking purposes. SPIONs without a magnetic field can improve the tissue repair productivity, be responsible for dynamic mechanical motivations for bone regeneration, encourage osteogenic differentiation of BMSCs, and develop bone healing in vivo (Santhosh and Ulrih 2013).

In a study, gelatin/SPION-scaffold were implanted in the incisor sockets of rat model which improved bone restoration in comparison to gelatin porous structure control without SPIONs. It is notable that the endocytic SPIONs stimulated the osteogenic and angiogenic performance of the cells result in better bone regeneration (Gu et al. 2013). The aggregation of SPIONs as a result of magnetic fields may change their biological impression. Definitely, decrease in cell uptake followed for the reason that agglomeration of the particles as a result of substantial variations in mutually the size and zeta potential. Furthermore, external magnetic fields may affect the biological properties of SPIONs such as therapeutic/toxic effects. In a research, (Fe^2+^/Fe^3+^)-doped HA (FeHA) nanoparticles in cultures with osteoblast-like cells in the absence, or presence, of an SMF were investigated. Application of external magnetic field to FeHA lead to a substantial cell growth, proliferation and more osteoblastic activity as a result of the tremendous biological impacts of HA and the partial iron content. Consequently, the variations in the biological characteristic and endocytosis of the cells, created by the MNPs using external magnetic field, may pointedly improve the cell performance and bone renewal abilities (Tampieri et al. 2014). Stem cells have excessive potential for tissue repair. Magnetically labeled cells have the potential application for bone tissue regeneration, containing cell targeting and cell patterning. SPIONs are able to operate as an ideal labeling and tracer tool for MSCs. MNP intake into the cells and make them manageable and manipulated by external magnetic field. In a study implantation of magnetically labeled MSCs were employed to regenerate serious chronic osteochondral traumas, exposure to an external magnetic field, considerably produced new chondrogenic tissues (Li et al. 2018).

Dip coating technique was used to fabricate magnetic HA/collagen scaffolds. The magnetic scaffolds support the attachment and proliferation of hBMSCs, and motivate osteoblastic differentiation. The results were along with a different study on MNP-HA magnetic scaffolds.

In another study, nanofibrous γ-Fe_2_O_3_/HA/polylactic acid was fabricated. This scaffold improved the proliferation of osteoblastic by reason of the SPION integration (Kim et al. 2006). In a current research, an injectable calcium phosphate/SPIONs cement has been developed by mixing with a SPION. Osteogenic differentiation and bone matrix mineral synthesis by the cells was similarly improved two folded in comparison to samples without SPIONs (Xia et al. 2018a). Fe_3_O_4_ nanoparticle/bioactive glass/polycaprolactone (Fe_3_O_4_/MBG/PCL) scaffolds was fabricated using a 3D printing method. The results indicate that cell growth on the Fe_3_O_4_/MBG/PCL scaffolds was greater than non-magnetized control sample (Zhang et al. 2014). In vivo results using rabbit model confirmed that the PCL/FeHA scaffolds were enhanced bone regeneration in comparison to non-magnetized control. SPIONs/nHA and PLA nanofibrous scaffold was implanted in the lumbar transverse defects in rabbit model and then SMF were applied. The MNP scaffold with application of an SMF persuaded more osteogenesis, new bone formation and remodeling in the rabbit defects (Hu et al. 2018). Along with stem cells and scaffolds, growth factors delivering are a vital method in bone tissue regeneration. MNPs have the potential for using as a delivery tool for biological mediators for instance drugs, chemotherapeutics, antibodies, peptide, oligonucleotides, and growth factors through magnetic fields. For example, gene delivery using MNPs possibly will be multifunctional, performing utilities that contain the identification, healing and visualization of the disease at the same time. Consequently, magnetic-based gene delivery is extremely promising method for stem cell therapy (McCarthy et al. 2007).

### Bone/biomaterials interface studies

For the effective integration of implants or scaffolds for tissue regeneration, cell adhesion to biomaterials is a vital necessity. Adjusting cells–scaffolds communications seems of most important to affect succeeding cell biological progressions for instance attachment, proliferation and differentiation (Tormos 2016). Numerous reports show that the adhesion of integrins in bone cells including osteoblasts and osteoclasts to the extracellular matrix is vital through the bone repair (Puleo and Nanci 1999). Bone extracellular matrix proteins arbitrate the biological function of cells through moderating their milieu. Motivation of the attachment, proliferation and differentiation of the bone cells are determined by the part of the superficial characteristics, chemical composition, electrostatic charge, texture, geometrical configuration, roughness and smoothness, of the replacement (Venkatesh and Sen 2017). The ceramic biomaterials may be abrasive and consequently, it is crucial to avoid them in uncontrolled damage neighboring to articular surfaces. Bioglass ionic extracts and surface exchanges stimulate the proliferation and differentiation of osteoblasts and the fabrication of the primary phenotypic biomarkers (Abid et al. 2017, 2016).

The cell activities are regulated by biodegradable polymers with properties such as chemical structure, polymer ratio of PLA or PGA for example, molecular weight and crystallinity. Polymer degradation products for instance catalysts, additives, byproducts and residual monomers that led to an inflammatory reaction and influence the cell attachment, cell survival and proliferation (Puppi et al. 2010). Composite structures present tolerable physiological and mechanical performance such as the characteristics and morphology of cortical and trabecular bone. Signaling factors can be included to bone composites to stimulate cell behavior and favor bone repair. Several factors impact on the release of growth factors, for example the surface charge and chemistry of composite, geometry, dimensions, porosity, wettability, crystallinity, the rate of degradation. The growth factor release could be regulated by diffusion, exterior motivation, enzymatic/chemical response (Muzzarelli 2011). The cell attachment to biomaterial and their following performances can be influenced by surface features for instance topography, hydrophobicity, charge, chemistry and special surface energy (Von Recum and Van Kooten 1996). These all affect the conformation, alignment and amounts of adhesion proteins including vitronectin or fibronectin that facilitate the interfaces among cells and biomaterial (Place et al. 2009). Lithography, colloidal particle adsorption, micro-contact printing, novel polymer preparations and self-assembled monolayers are all employed to analyze the interactions among cells and biomaterials at the micro- and nanometer scale (Ma et al. 2007). These methods may be utilized to regulate the topographic properties, micrometer or nanometer ridges, grooves pits, islands, holes. Some studies have revealed improved bone–biomaterial interactions with a high surface roughness. In a study, PLA-polystyrene films with porous of about 45 nm caused human fetal osteoblasts to proliferate expressively more and attach greatly better than a flat PLA surface (Kochesfahani 2016). Current investigations have employed in vitro self-assembling monolayers containing PEG, OH, COOH, NH_2_ and CH_3_ groups to assess the consequence of surface chemistry and hydrophilicity on protein adsorption and cell performance such as the attachment strength of MC3T3-E1 preosteoblasts and the medium mineralization. The results show mineralization by cells on OH and NH_2_ surfaces is associated with improved alpha 5 beta 1 integrin adhesion and FAK stimulation (Keselowsky et al. 2003). Besides, the experiments approved that osteoblasts adhered and proliferated further on positively charged hydrogels in comparison to neutral or negatively charged ones (Liu et al. 2014).

## Conclusions

This review investigated the bone physiology, different strategies for bone repair and interface studies. The published results provide insightful information for development of new biomaterial-based products. However, there is a vital necessity to investigate the results of all clinical trials. There are several bone repair strategies including bone graft replacements, implantable materials and scaffolds, optimized 3D structure and favored surface properties. Bioactive constituents are necessitated while the bone defects become larger. For this goal, signaling factors, polypeptides and small biomolecules are presently being assessed at the pre-clinical phase. The incorporation of bioactive molecules with novel carriers may result in a developed conveyance of the active molecules at a reliable and useful amount. Besides, cell-based approaches can be utilized for big and complicated bone defects. The efficient clinical treatment demands more controlling phases for future developments.
